# Constriction Band Release in a Neonatal Ischemic Limb: A Pediatric Anesthesiology Case Report

**DOI:** 10.7759/cureus.36895

**Published:** 2023-03-30

**Authors:** James Paul, Taylor Kaiser, Delaney A Dalldorf, Brian Chenoweth, Eddison Williams, Joseph M Sisk

**Affiliations:** 1 Anesthesiology, Emory University, Atlanta, USA; 2 Anesthesiology, University of Oklahoma Health Sciences Center, Oklahoma City, USA; 3 Anesthesiology, University of North Carolina at Chapel Hill, Chapel Hill, USA; 4 Orthopedic Surgery, University of Oklahoma Health Sciences Center, Oklahoma City, USA; 5 Anesthesiology, Tufts University School of Medicine, Boston, USA

**Keywords:** pediatric anesthesiology, fasciotomy, post-reperfusion syndrome, neonatal limb ischemia, constriction band syndrome

## Abstract

A 1.1 Kg, four-hour-old, 29-weeks-gestation male patient presented with right upper extremity ischemia secondary to neonatal constriction band syndrome. Emergency constriction band release was deemed necessary to facilitate limb salvage. The anesthetic management of this patient required close communication with the surgical team and meticulous attention to the risks of post-reperfusion syndrome and blood loss in this fragile neonate. Limb salvage was ultimately successful, and the patient demonstrated full neurologic recovery at his two-year follow-up visit.

## Introduction

Limb ischemia in the neonatal period is a rare complication of constriction band syndrome (CBS) and may cause irreversible and devastating injuries. The syndrome has been referred to under many names and attributed to several causes. However, fibrous bands presenting within the first hours of life with associated vascular and neuronal compromise is a paramount diagnostic finding of CBS [1-2]. The pathogenesis is divided into extrinsic and intrinsic models. Common extrinsic theories include amniotic bands, umbilical cord loops, and oligohydramnios [1-2]. Streeter dysplasia, a proposed intrinsic theory, suggests disruption of early embryonic development as the cause of constriction band formation. Despite various theories, the syndrome's etiology remains complex and multifactorial [3].

Management of the ischemic limb in the neonate is focused on the salvage of impending tissue loss while preserving function and development. Delay in diagnosis and thus in surgical intervention has been associated with poor prognoses including amputation, infection, and contracture [2]. The anesthetic considerations of neonatal limb ischemia are focused on minimizing the physiologic impact of the toxic metabolites released into circulation when the constriction band is released and managing the potential blood loss associated with fasciotomies. These challenges may be further exacerbated by a preterm neonate’s low body weight and overall frailty. The maintenance of effective ventilation and adequate perfusion during the physiologic insult of band-release surgery are critical aspects of the anesthetic care of these patients [4]. 

Neonatal CBS is explored in the surgical literature, but the anesthetic management of these patients is not well described. The objective of this case report is to discuss the time-sensitive nature of the surgical treatment of a neonatal ischemic limb and the difficulties associated with anesthetic care of these patients with a focus on avoiding post-reperfusion syndrome and hemodynamic collapse. This manuscript adheres to the applicable Enhancing the QUAlity and Transparency Of health Research (EQUATOR) guideline. Written Health Insurance Portability and Accountability Act authorization were obtained as well as consent for the publication of photographs.

## Case presentation

**Figure 1:**
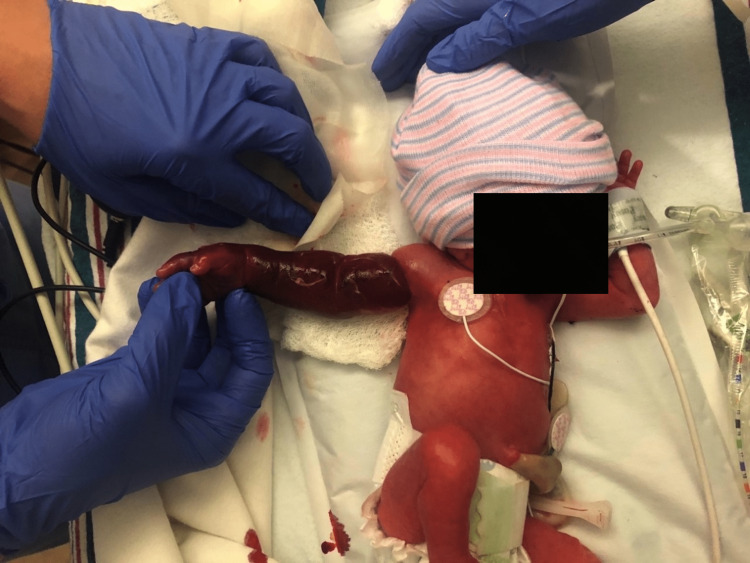
Neonate immediately after birth with a constriction band on the right upper extremity causing limb ischemia.

A 1.1 Kg, four-hour-old, 29-weeks-gestation male patient presented with a constriction band on the right upper arm following fetal distress in the setting of twin gestation. Upon delivery, the affected arm showed evidence of ischemia due to band compression of the vascular and nervous tissue in the arm (Figure 1). Pulses were detected using doppler sonography; however, the constriction band completely obstructed venous outflow. The orthopedic hand surgery team deemed urgent intervention was needed to avoid amputation and scheduled the patient for urgent release of his constriction band.

The patient was intubated for respiratory distress at the time of delivery and remained stable on controlled mechanical ventilation in pressure control mode with the following settings: peak inspiratory pressure 12, positive end-expiratory pressure 6, rate 30, and inspired oxygen 30%. The neonatology team obtained intravenous (IV) access in the form of an umbilical vein catheter (UVC) and two 24-gauge peripheral IVs. The patient was maintained on a dextrose-containing fluid infusion for the prevention of hypoglycemia. A non-invasive cuff was utilized for blood pressure monitoring. Congenital heart disease was suspected prenatally; however, a preoperative echo demonstrated transitional circulation and no structural anomalies. No additional congenital anomalies were identified in the patient or his twin. Informed consent for general anesthesia and blood product administration was obtained. In anticipation of intraoperative transfusion, 30 mL/Kg of packed red blood cells were prepared and delivered to the operating room. Multiple weight-based doses of code medications were prepared and emergency airway equipment was readily available during patient transport. 

With the assistance of a neonatal intensive care unit (NICU) respiratory therapist, the patient was ventilated during transport to the operating room using his NICU ventilator. Upon arrival in the operating room, the patient was connected to standard American Society of Anesthesiologists monitors and the NICU ventilator was continued on the above ventilator settings. Pre-op vitals included a SpO2 of 99%, a heart rate (HR) of 164, and a non-invasive blood pressure (NIBP) of 44/20 with a mean arterial pressure (MAP) of 28. Anesthesia was maintained with a combination of fentanyl (3 mcg) and rocuronium (7 mg) administered in divided doses throughout the case. At the time of surgery, an umbilical artery catheter had not been placed. Given the concern for possible hemodynamic volatility and the need for lab monitoring, a left radial arterial line was attempted with ultrasound guidance but was unsuccessful. Rather than further delay in operative intervention and risk of vascular injury to the contralateral arm, the decision was made to NIBP cuff readings and to use the patient’s UVC to draw labs for point of care (POC) testing. The patient’s baseline venous blood gas (VBG) showed a pH of 7.35, a PCO2 of 43, a PO2 of 42 with a base excess of -2 mEq/L, and a bicarbonate of 24 mEq/L. The lab also demonstrated a baseline potassium (K) of 3.1 mEq/L and an ionized calcium (iCa) of 1.4 mmol/L. Preoperative hemoglobin (Hgb) was 10.5 g/dL with a hematocrit (HCT) of 31%.

**Figure 2:**
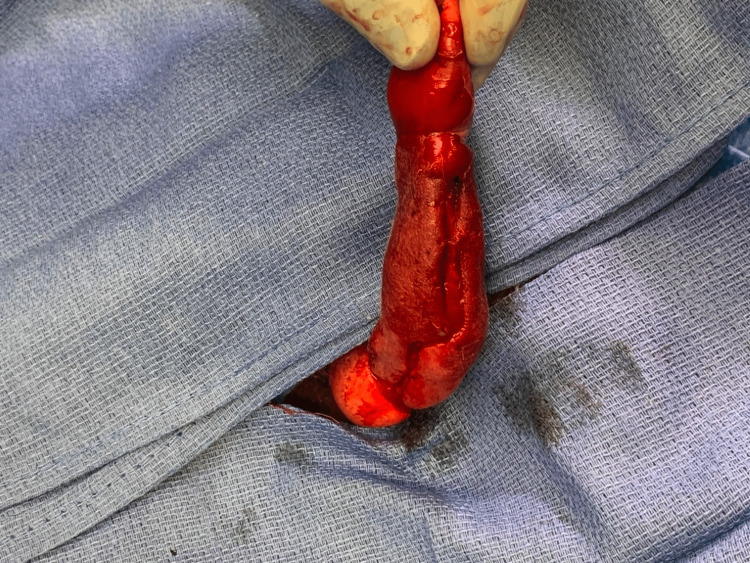
Right upper extremity following constriction band release and anterior and poster compartment fasciotomies.

Given the concern that constriction band release could disseminate significant quantities of anaerobic metabolites, inflammatory mediators, and potassium into the body, the band was gradually released over several minutes to mitigate the risk of post-reperfusion syndrome, arrhythmias, and hemodynamic collapse. The anesthesia team communicated with the surgical team closely during band release to ensure the patient had the opportunity to adapt to the influx of vasoactive mediators. Fortunately, the patient tolerated reperfusion well with minimal change in his hemodynamic status. Once the amniotic band was completely released, the presence of pulses in the radial and ulnar arteries was confirmed with doppler sonography. Fasciotomies were performed on the anterior and posterior compartments of the arm (Figure 2). Following fasciotomy, a VBG demonstrated a pH of 7.22, a PCO2 of 60, a PO2 of 47 with a base excess of -3 mEq/L, and a bicarbonate of 25 mEq/L. Post-release, iCa was 1.3 mmol/L with a K of 3.3 mEq/L. Hgb was 8.2 g/dL with an HCT of 24%. Given the patient’s estimated blood volume of 110 mL, 30 mL of blood was transfused during the fasciotomies. Intraoperative blood loss was estimated to be 20 mL upon completion of the procedure.

**Figure 3:**
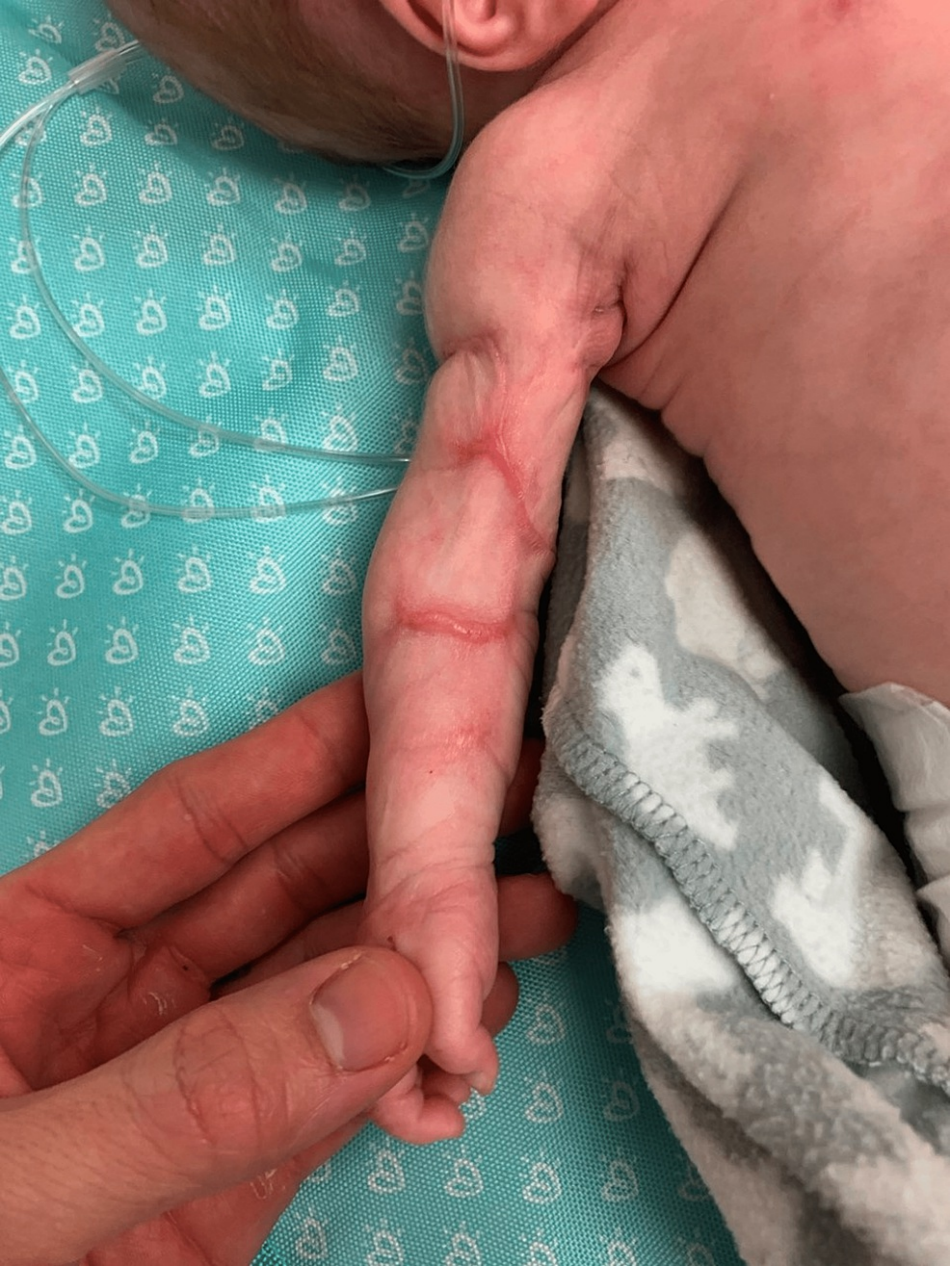
Infant at six months of age showing the successfully salvaged right upper extremity.

**Figure 4:**
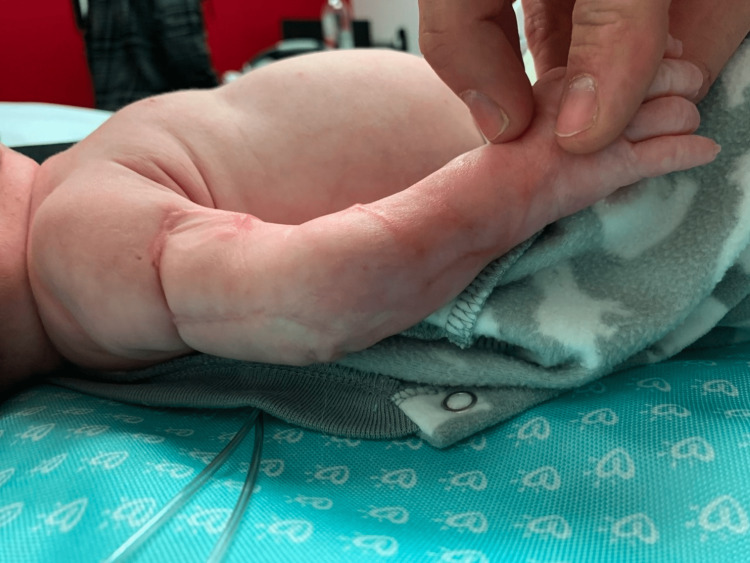
Infant at six months of age showing the successfully salvaged right upper extremity.

After the release, the patient was continued on mechanical ventilation and was transported back to the NICU in stable condition. Post-op vitals were a SpO2 of 99%, HR 149, NIBP 44/20, and MAP 28. Post-op labs showed a Hgb of 12.6, HCT of 27, K of 3.4, and an iCa of 1.31 mmol/L. He was successfully extubated several days later, ultimately requiring continuous supplemental oxygen for bronchopulmonary dysplasia. At two years of age, the patient demonstrated full neurologic recovery. The successfully salvaged limb at six months of age is shown in Figures 3-4.

## Discussion

Anesthesia considerations for this case included the young gestational age of the neonate and his underdeveloped organs, the ramifications of reperfusion of the ischemic limb, and the potential blood loss associated with fasciotomies. In the setting of the patient’s prematurity, we considered that the underdeveloped lungs and insufficient surfactant production could pose a challenge for oxygenation and ventilation. Intraoperative management balanced the risks of positive pressure ventilation on immature lungs and hyperoxia in a preterm neonate with the need to ensure adequate oxygenation, ventilation, and perfusion of our patient [4]. In the management of our patient, 30% inspired oxygen was used in mechanical ventilation in keeping with the patient’s NICU ventilator settings.

Reperfusion of ischemic tissue washes metabolic waste products and inflammatory molecules into the systemic circulation which can cause multiple complications, including hemodynamic collapse [5]. There is little literature describing the hemodynamic response to limb reperfusion in premature neonates; however, cardiac arrest following limb reperfusion has been described in adult patients [6]. While our patient ultimately tolerated the procedure well, judicious caution was appropriate. Neonates have fewer compensatory mechanisms and less tolerance for these stressors than older patients. These patients are particularly sensitive to hypocalcemia. Our patient was empirically treated with 30 mg/Kg calcium gluconate prior to reperfusion. Supplemental calcium in the setting of post-reperfusion syndrome is important as it functions to stabilize the myocardium in the event of hyperkalemia when the metabolites are reintroduced from the hypo-perfused limb. In addition to stabilizing the myocardium, the administration of calcium gluconate has the secondary benefit of preventing hypocalcemia due to the binding of calcium to the citrate found in packed red blood cells. Excellent communication between the surgical and anesthesia teams ensured the constriction band was released gradually enough to allow the anesthesia team to respond to the physiologic insult. 

Lastly, fasciotomies may result in significant blood loss as the compartments are opened to allow the ischemic tissue and cytotoxic edema to swell outside the normal confines of the limb [7-8]. While transfusion may help manage this, the risks of transfusion in preterm neonates include cardiac overload and acute lung injury [9-10]. Given this 1.1 Kg neonate had an estimated blood volume of only 110 mL, seemingly small amounts of blood loss had the potential to be catastrophic. Early transfusion was beneficial in preserving the hemodynamic status of our patient as he endured the stressors associated with limb reperfusion.

## Conclusions

This case report highlights the challenging management of a premature neonate with an ischemic limb. Limb reperfusion may have disastrous hemodynamic consequences due to the lack of physiologic reserve and electrolyte imbalance. Clear communication with the surgical team to facilitate the controlled release of the constriction band, close attention to electrolyte homeostasis, and early transfusion were all critical elements of the successful anesthetic care of this patient.
